# Low occurrence of MRI spinal changes in elite climbing athletes; a cross-sectional study

**DOI:** 10.1186/s13102-023-00637-z

**Published:** 2023-03-09

**Authors:** Fredrik Identeg, Kerstin Lagerstrand, Henrik Hedelin, Eric Hamrin Senorski, Mikael Sansone, Hanna Hebelka

**Affiliations:** 1 Department of Orthopaedics, Gothenburg, Sweden; 2 Department of Radiology, Sahlgrenska University Hospital, Gothenburg, Sweden; 3 Department of Medical Physics and Biomedical Engineering, Sahlgrenska University Hospital, Gothenburg, Sweden; 4 Department of Health and Rehabilitation, Institute of Neuroscience and Physiology, University of Gothenburg, Gothenburg, Sweden; 5 Institute of Clinical Sciences, Sahlgrenska Academy, University of Gothenburg, Gothenburg, Sweden

**Keywords:** Rock-climbing, Back pain, Magnetic resonance imaging, Thoracic spine, Lumbar spine, Disc degeneration, Endplate

## Abstract

**Objectives:**

To examine the occurrence of magnetic resonance imaging (MRI) changes in the thoracolumbar spine among elite climbing athletes.

**Methods:**

All climbers of the Swedish national sport climbing team (n = 8), and individuals having trained for selection to the national team (n = 11), were prospectively included. A control group, matched in age and sex, were recruited. All participants underwent a thoracolumbar MRI (1.5 T, T1- and T2-weighted imaging), evaluated according to Pfirrmann classification, modified Endplate defect score, Modic changes, apophyseal injuries and spondylolisthesis. Pfirrmann ≥ 3, Endplate defect score ≥ 2 and Modic ≥ 1 was defined as degenerative findings.

**Results:**

Fifteen individuals, 8 women, participated in both the climbing group (mean age 23.1, SD 3.2 years) and the control group respectively (mean age 24.3, SD 1.5 years). In the climbing group, 6.1% of the thoracic and 10.6% of the lumbar intervertebral discs showed signs of degeneration according to Pfirrmann. One disc with a grade above 3 was present. Modic changes in the thoracic/lumbar spine were prevalent in 1.7%/1.3% of the vertebrae. Degenerative endplate changes according to the Endplate defect score were found in 8.9% and 6.6% of the thoracic and lumbar spinal segments of the climbing group, respectively. Two apophyseal injuries were found, while no participants displayed signs of spondylolisthesis. There was no difference in point-prevalence of radiographic spinal changes between climbers and controls (0.07 < p < 1.0).

**Conclusion:**

In this small cross-sectional study, only a low proportion of elite climbers displayed changes of the spinal endplates or intervertebral discs, as opposed to other sports with high spinal loads. Most observed abnormalities were low grade degenerative changes and did not differ statistically compared to controls.

## Introduction

In recent decades, climbing has evolved from being a niche activity for outdoor enthusiasts to a widely popular sport, which is now easily accessible to people all over the world. The inclusion of climbing in the Olympics of 2020, and the rapid increase of climbing competitions, highlights the trend of climbing evolving into a competitive sport [1]. This shift is likely to result in more rigorous training regimes and calls to attention the potential injury patterns associated with intense training for performance climbing.

The athlete’s spine is subject to frequent and considerable load, which naturally increases with the level of sport participation and training [2, 3]. Radiographic changes of the spine have repeatedly been reported to be more prevalent in certain groups of athletes compared to controls [4–10]. Examples of such changes include disc degeneration, disc herniation, apophyseal ring injury, spondylolysis, and spondylolisthesis. Although the etiology of back pain is still not completely understood, such spinal changes have been associated with thoracolumbar back pain [3, 11, 12].

While climbing consists of many different sub-disciplines with varying loading patterns, indoor bouldering is a probably the most frequent training method for high-performance climbers. This training method repeatedly place high axial loads on the spine when jumping or falling from up to five meters of climbing, landing on padded mats. High compressional loads in flexion and extension of the spine are also frequent during climbing, regardless of climbing discipline. Ex vivo models have suggested that these specific loads generate degenerative changes of the intervertebral discs (IVD) and vertebral endplates [13–15].

Considering the high prevalence of radiographic spinal changes in elite athletes of sports with similar spinal loading patterns, elite climbers could be expected to be at risk of developing early disc degeneration and other structural changes. However, the subject is currently unexplored, and the occurrence of radiographic spinal changes in climbers has, to date, not been described. The aim of this study was to examine the occurrence of spinal changes on magnetic resonance imaging (MRI) among elite climbing athletes.

## Methods

### Participants

All climbers (bouldering and sport climbing) of the Swedish senior national sport climbing team were prospectively invited to participate (n = 8). In addition, due to the limited sample size of the national team, the head manager of the Swedish national climbing team invited additional climbers, meeting the inclusion criteria of high-level performance (n = 11). The participants were either currently training to participate in the national team or had previously been training to participate in the team.

Since high cumulative training load and pre-growth spurt training debut are plausible risk factors for developing spinal changes, inclusion criteria were based on a combination of criteria where climbers had been exposed to these plausible risks. Inclusion criteria of all climbing participants were: a minimum climbing level of elite during last 12 months, as classified by the International Rock Climbing Research Association (IRCRA) [18], age over 18 years, having a minimum of five years of climbing experience, current or previous participation in national or international climbing competitions and frequent use of bouldering as a training method. Since elite-level climbers already constitute a limited study sample, the inclusion was not limited by an upper age limit, nor was back pain included as a parameter in the inclusion criteria.

A control group was recruited, matched in terms of age and sex, through advertisement on social media. Participants voluntarily reported their interest to participate in the study. For the control group, any experience of regular climbing, as well as previous or present participation on elite level, in any sport, led to exclusion. Individuals that had tried climbing on a few occasions were allowed to participate. Exclusion criteria for all participants were prior spinal surgery, and contraindications to undergo MRI.

### Demographics and back pain questionnaires

An electronic survey was used to gather information on weight and height in order to calculate each participant’s Body Mass Index (BMI). Climbing level for the last 12 months was collected and classified according to the recommendations of the IRCRA [18]. The prevalence of jumping descent in bouldering was recorded through questions designed by the authors.

The Nordic questionnaire of back pain [19], adjusted for sport specific settings [20], was used to examine the lifetime and one-year prevalence of thoracolumbar back pain as well as training volume of the participants. The Nordic questionnaire of back pain has shown acceptable test–retest reliability and validity to clinical examination [19, 20]. Based on the sport adjusted Nordic questionnaire, questions focusing on training volume between 10 and 20 years of age were computed, since athletes are plausibly more susceptible to develop radiographic spinal changes of the spine during the growth spurt [13, 21]. The Oswestry back pain disability index [22] was used to examine disability associated to thoracic and lumbar back pain.

### MRI examination

Thoracolumbar spinal MRI examinations were performed at the Department of Radiology, Carlanderska Hospital using a 1.5 T scanner (Signa, GE Healthcare, Chicago, IL, USA). The MRI protocol included sagittal T1-and T2-weighted sequences (Th1-S1). In the thoracic spine, a field of view of 360 × 360mm^2^ and slice thickness of 3 mm was used. In the lumbar spine a field of view of 320 × 320mm^2^ and slice thickness of 3.5 mm was utilized.

The MRI images were classified by a senior radiologist (> 15 years of experience) according to a predetermined standardized protocol. Disc degeneration was classified according to the Pfirrmann classification [23]. In the thoracic spine, no distinction between Pfirrmann grade 1 and grade 2 was made since the resolution of the images was not considered adequate for reliable differentiation between these grades. Vertebral and endplate changes were classified according to the Modic classification [24] and a modified Endplate defect score, adapted to our MRI protocol. The Endplate defect score [25] was modified where Type I-III (representing no degeneration) were pooled (Table 1). Schmorl’s nodes were classified as present or not present and defined as a vertebral endplate irregularity associated with intraspongious disc herniation, irrespective of the size, at either the cranial or caudal endplate, or at both endplates relative to the lumbar disc level. Spondylolisthesis was assessed as either present or not [26, 27]. Similarly, vertebral apophyseal injury, defined as any irregularity or signal changes in the apophyseal region, was categorized as either present or not.

**Table 1:** Modified endplate score, based on the original endplate defect score [25]

Modified endplate defect score	Original endplate defect score
1	Type I—Normal endplate with no interruption
	Type II—Thinning of the endplate, no obvious break
	Type III—Focal endplate defect with established disc marrow contact but with maintained endplate contour
2	Type IV—Endplate defects < 25% of the endplate area
3	Type V—Endplate defects up to 50% of the endplate area
4	Type VI—Extensive damaged endplates up to total destruction

Intra-observer and inter-observer reliability measures were carried out on a set of 15 individuals (5 of the climbers and 10 back pain patients not included in the current study) by the senior radiologist and an additional radiologist (5 years of experience). The latter repeated the evaluation after one month, blinded to previous result.

### Statistical analysis

The IBM SPSS Statistics for MAC, version 24 (IBM Corp., Armonk, N.Y., USA) was used for demographic description of the data and statistical tests were performed using the statistical analysis software SAS 9.4 for Windows (SAS Institute Inc., Cary, NC, USA). The level of significance was set at p < 0.05.

For inference testing of spinal parameters, all non-dichotomous spinal parameters were dichotomized according to the established cut off for degenerative findings for each classification system (Pfirrmann ≥ 3, Endplate defect score ≥ 2, Modic ≥ 1).

For comparison of training amount, thoracolumbar back pain, and spinal changes between groups; Fisher ´s Exact test (lowest 1-sided p-value multiplied by 2) was used for dichotomous variables and the Mantel–Haenszel Chi Square Exact test was used for ordered categorical variables. The Fisher ´s Non-Parametric Permutation Test was used for continuous variables. The cumulative number of IVDs/levels/vertebrae per participant were compared between the climbing and control group.

Intra-observer and inter-observer reliability measures were analyzed with Gwent’s agreement coefficient with type 1 utilized for nominal and dichotomous variables and type 2 for ordinal variables.

## Results

### Demographics

A total of 15 individuals (8 women and 7 men) participated in each study group. Two climbers from the national team, and two additional climbers declined participation. None of the eligible participants were excluded. The mean age of the climbing group was 23.1 years (3.2) and of the control group 24.3 (1.5) years. The mean BMI was 20.6 (3) for the climbing group, and 23.2 (1.7) for the controls (Table 2). Apart from differences in weight (p = 0.04) and BMI (p < 0.001) between groups, there were no other demographic differences between the climbing group and the control group.

**Table 2:** Group characteristics of climbers and controls

	Climbers n = 15	Controls n = 15	Mean difference between groups	*p*-value
Sex				
Female	8 (53.3)	8 (53.3)	0 (− 37.4; 37.4)	1.00
Male	7 (46.7)	7 (46.7)	0 (− 37.4; 37.4)	1.00
Age (years)	23.1 (3.2)	24.3 (1.5)	− 1.3 (− 3.2; 0.6)	0.20
Height (cm)	171.0 (7.9)	171.0 (7.0)	0.0 (− 5.7; 5.7)	1.00
Weight (kg)	60.7 (10.5)	68.1 (7.1)	− 7.3 (− 14.2; − 0.4)	0.04*
BMI (kg/m2)	20.6 (3)	23.2 (1.7)	− 2.6 (− 4.0; − 1.2)	< 0.001*

For categorical variables n (%) is presented. For continuous normally distributed variables, Mean (SD) / (95% CI for Mean difference between group) is presented. *P*-values that are statistically significant are labeled as *

### Training habits

The climbing group had been climbing actively for a mean (SD) of 12 years (5), and bouldering for 11 (5) years. Eighty-seven percent of the climbing participants had a yearly training volume of over 400 h and 47% had a yearly training volume above 700 h. Seventy-three percent of the climbers had a yearly bouldering volume of above 400 h. The mean (SD) bouldering level of the participants was IRCRA 23.75 (5) (7C Font). Eighty-six percent of the climbing participants reported that descending from boulders through jumping occurred in more than 50% of the descents. A statistical difference in training volume between the climbing and control group was found for the current year and for the training volume between 16 and 20 years of age (p = 0.05, 0.001) (Table 3).

**Table 3:** Description of training habits of climbers and controls

	Climbers n = 15	Controls n = 15	*p*-value
Maximum climbing level IRCRA	23.75 (5)	–	
Years climbing (climbers)/Years training (controls)	12 (5)	14 (7)	
Years bouldering	11 (5)	–	
Yearly training volume			0.05*
< 400 h	2 (13)	8 (53)	
400–700 h	6 (40)	7 (47)	
> 700 h	7 (47)	–	
Yearly training volume age 10–15 years			0.55
< 400 h	6 (40)	7 (47)	
400–700 h	7 (47)	8 (53)	
> 700 h	2 (13)	–	
Yearly training volume age 16–20 years			0.001*
< 400 h	3 (20)	3 (20)	
400–700 h	4 (27)	12 (80)	
> 700 h	8 (53)	–	
Yearly bouldering volume			
< 400 h	4 (27)	–	
400–700 h	8 (53)	–	
> 700 h	3 (20)	–	
Frequency of jumping bouldering descent			
Always	–	–	
Almost always	5 (33)	–	
Most often	5 (33)	–	
Half of the times	3 (20)	–	
Seldomly	2 (13)	–	
Close to never/Never	–	–	

For categorical variables n (%) is presented. For continuous normally distributed variables Mean (SD) is presented. *P*-values that are statistically significant are labeled as *

The control group reported having trained regularly for a mean (SD) of 14 years (7). In the control group, 47% reported training above 400 h per year and 0% had a yearly training volume of above 700 h (Table 3).

### Thoracolumbar back pain

In the climbing group, 33% reported ever having experienced thoracic back pain as compared to 13% in the control group (p = 0.39). The lifetime prevalence of lumbar back pain was 73% in the climbing group and 60% in the control group (p = 0.7). The yearly prevalence of thoracic and lumbar back pain among climbers was 33% and 60%, in comparison to 13% and 53% in the control group. There were no differences in the lifetime or yearly prevalence of thoracic and lumbar back pain between climbers and controls (Table 4). No significant difference between the groups were found in Oswestry disability Index, (p = 0.5) (Table 4).

**Table 4:** Occurrence of thoracolumbar back pain in climbers and controls

	Climbers n = 15	Controls n = 15	Difference between groups	*P*-value
Ever experienced thoracic back pain				0.39
Yes	5 (33)	2 (13)	20.0 (− 12.4; 50.3)	
No	10 (67)	13 (87)	− 20.0 (− 50.3; 12.4)	
Days experienced thoracic back pain last year				0.64
0 days	–	–		
1–7 days	2 (40)	1 (50)		
8–30 days	3 (60)	–		
More than 30 days	–	1 (50.0)		
Ever Experienced lumbar back pain				0.70
Yes	11 (73)	9 (60)	13.3 (− 21.7; 46.3)	
No	4 (27)	6 (40)	− 13.3 (− 46.3; 21.7)	
Days experience lumbar back pain last year				0.66
0 days	2 (18)	1 (11)		
1–7 days	3 (27)	6 (67)		
8–30 days	4 (37)	–		
More than 30 days	2 (18)	2 (22)		
Oswestry disability index—total score	2.7 (3.1)	4.0 (6.4)	− 1.3 (− 5.1; 2.2)	0.50

For dichotomous/categorical variables n (%) is presented

For continuous normally distributed variables Mean (SD) / (95% CI for mean difference between group) is presented

### Spinal changes

**Fig. 1:**
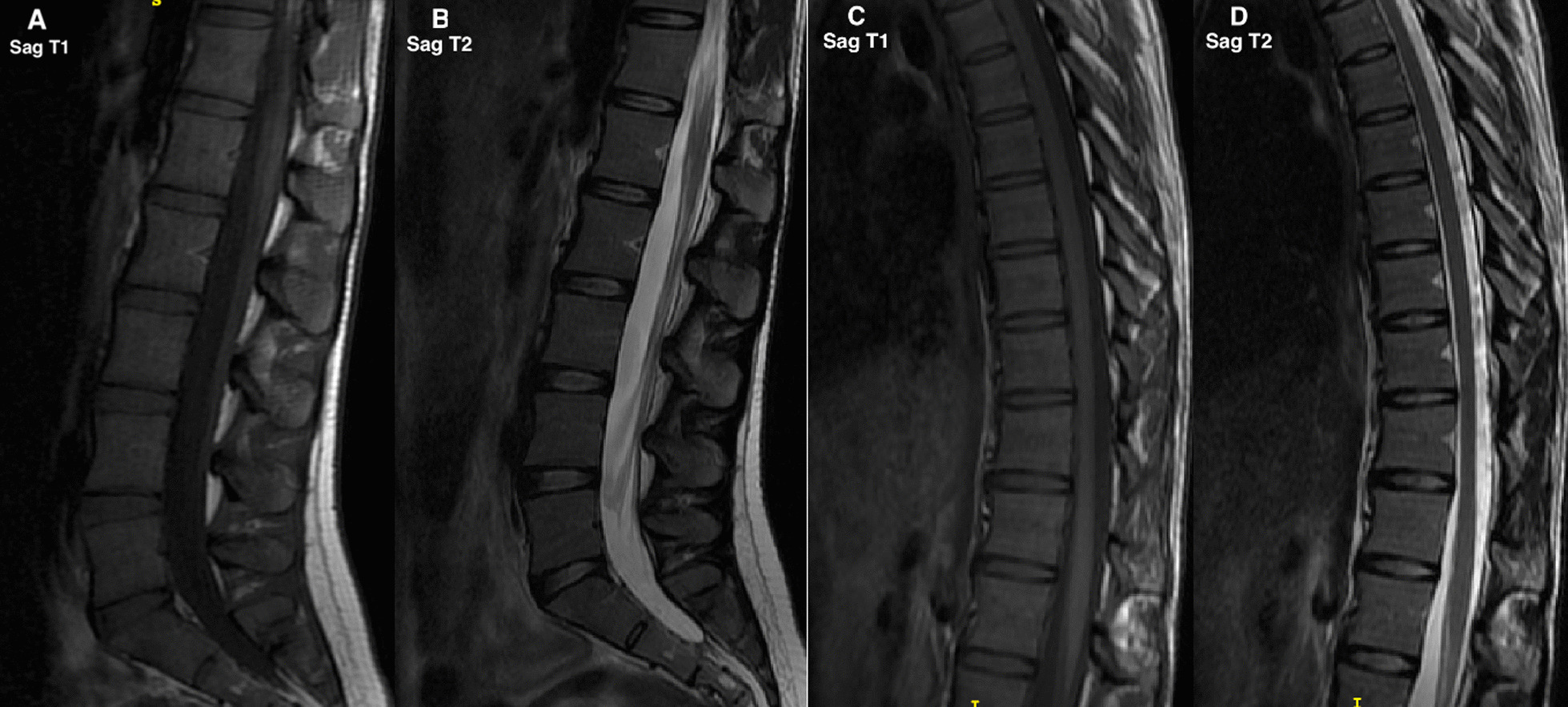
MRI of a female climber in the 20s with few prevalent spinal changes. The lumbar and thoracic spine (A–D) show few signs of spinal changes of the endplates and IVDs

**Fig. 2:**
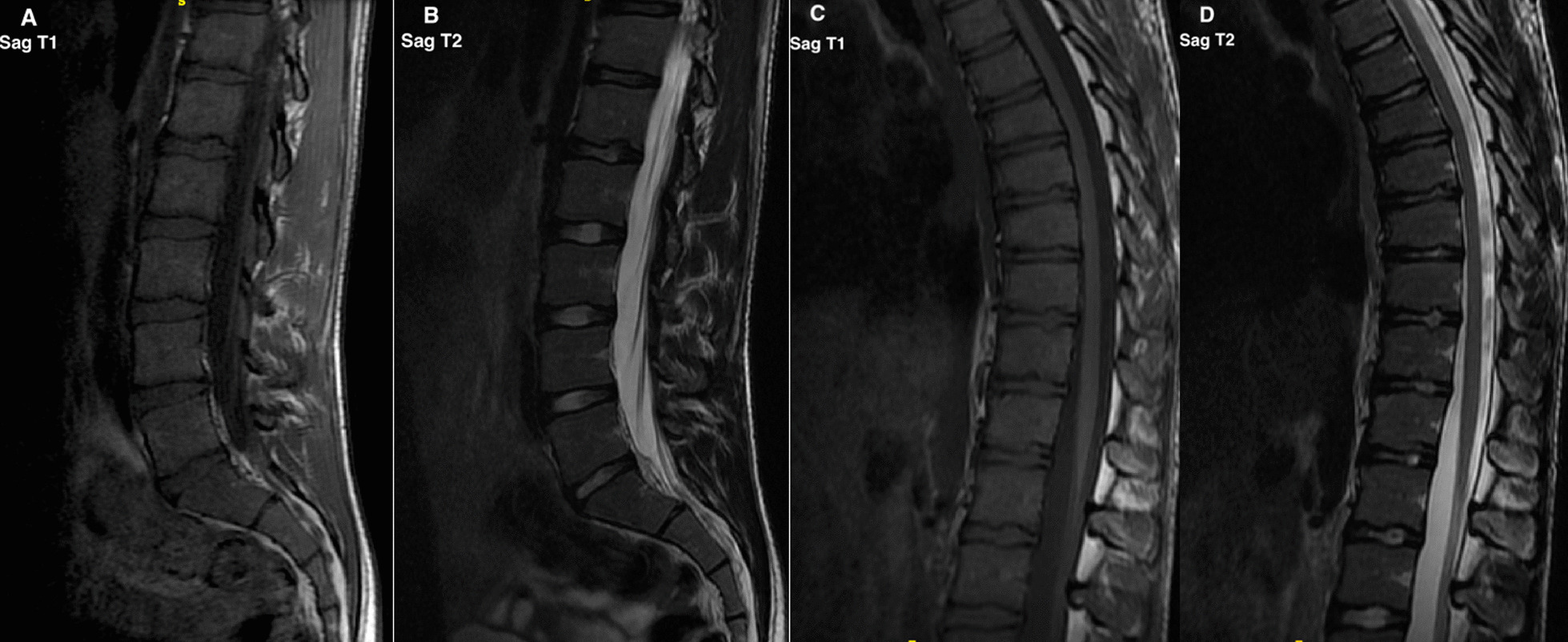
MRI of a male climber in the 20s, with the highest accumulated number of spinal changes of the climbing participants. The lumbar imaging (A + B) displays endplate changes of the Th12-L1 segment. The thoracic imaging (C + D) displays multiple spinal segments with low grade endplate changes and reduced disc signal intensity. This individual constituted an outlier in the sample, with endplate irregularities commonly seen in Scheuermann´s disease. The participant did not however fulfil the criteria for Scheuermann´s disease

In the climbing group, 11 of 180 IVDs (6.1%) in the thoracic spine were of Pfirrmann grade 3. No IVDs above grade 3 were present. One segment displayed Modic Type 1 changes, and two segments had Modic Type 2 changes. According to the modified Endplate defect score, 11 of 180 IVDs (6.1%) had adjacent endplates with score 2, and five levels (2.8%) were scored 3. No levels had an Endplate defect score above 3 (Table 5). Examples of participants with a high, and low number of spinal changes, are displayed in Fig. 1 and Fig. 2.

**Table 5:** Cumulative number of discs in the thoracolumbar spine (Th1-S1) for each classification system

Thoracic spine	Lumbar spine				
	Climbers n = 180	Controls n = 180		Climbers n = 75	Controls n = 75
Pfirrmann					
–	–	–	1	24 (32.0)	26 (34.7)
1–2	169 (93.9)	149 (82.8)	2	43 (57.4)	34 (45.3)
3	11 (6.1)	27 (15)	3	7 (9.3)	14 (18.7)
4	–	4 (2.2)	4	1 (1.3)	1 (1.3)
5	–	–	5	–	–
Total	180 (100)	180 (100)	Total	75 (100)	75 (100)
Modic					
0	177 (98.3)	179 (99.4)	0	74 (98.7)	74 (98.7)
1	1 (0.6)	–	1	1 (1.3)	–
2	2 (1.1)	–	2	–	1 (1.3)
3	–	1 (0.6)	3	–	–
Total	180 (100)	180 (100)	Total	75 (100)	75 (100)
Modified Endplate defect score					
1	164 (91.1)	154 (85.6)	1	70 (93.4)	65 (86.7)
2	11 (6.1)	20 (11.1)	2	4 (5.3)	10 (13.3)
3	5 (2.8)	6 (3.3)	3	1 (1.3)	0
4	–	–	4	–	–
Total	180 (100)	180 (100)	Total	75 (100)	75 (100)

Number of IVDs/levels per grade are presented

For dichotomous/categorical variables n (%) is presented

In the climbing group, 43 out of 75 IVDs (57.4%) in the lumbar spine were of Pfirrmann grade 2, seven IVDs (9.3%) grade 3 and one IVD of grade 4. One out of 75 segments displayed Modic Type 1 changes, and no Type 2 or Type 3 changes were found. According to the modified Endplate defect score, four out of 75 IVDs (5.3%) had adjacent endplates scored as 2 and one level scored as 3. No endplates were of score 4

Two apophyseal injuries were found in the climbing group, one in the thoracic spine, and one in the lumbar spine. No cases of spondylolisthesis were displayed in the climbing group (Table 6). Corresponding spinal changes for the control group for the thoracic and the lumbar spine, are reported in Table 6.

There were no statistically significant differences between the climbing and control group in any of the recorded parameters in the thoracic and lumbar spine, when comparing the cumulative number of spine changes per participant (Table 6).

**Table 6:** Comparison of cumulative number of spinal changes in the thoracic and lumbar spine between climbers and controls (Th1-S1)

Thoracic spinal changes	Lumbar spinal changes						
	Climbers	Controls	*p*-value		Climbers	Controls	*p*-value
	n = 15	n = 15			n = 15	n = 15	
Pfirrmann - N of IVDs per participant with grade ≥ 3							
0	11 (73)	5 (33.3)	0.07	0	9 (60)	5 (33)	0.18
1	1 (7)	3 (20.0)		1	4 (27)	5 (33)	
2	-	1 (6.7)		2	2 (13)	5 (33)	
3	2 (13)	2 (13.3)		3	–	–	
4	1 (7)	1 (6.7)		4	–	–	
5	–	2 (13.3)		5	–	–	
6	–	1 (6.7)		6	–	–	
Modified Endplate defect score - N of levels per participant with grade ≥ 2							
0	9 (60)	3 (20.0)	0.38	0	12 (80)	9 (60)	0.43
1	2 (13)	6 (40.0)		1	2 (13)	3 (20)	
2	2 (13)	2 (13.3)		2	-	2 (13)	
3	1 (7)	2 (13.3)		3	1 (7)	1 (7)	
4	-	1 (6.7)		4	–	–	
6	-	1 (6.7)		5	–	–	
7	1 (7)	–		6	–	–	
Modic - N of levels per participant with grade ≥ 1							
0	12 (80)	14 (93.3)	0.6	0	14 (93)	14 (93)	1.00
1	3 (20)	1 (6.7)		1	1 (7)	1 (7)	
Schmorl's Nodes - N of vertebrae per participant with positive findings							
0	13 (86)	9 (60)	0.38	0	11 (73)	11 (73)	1.00
1	1 (7)	2 (13)		1	3 (20)	2 (13)	
2	–	2 (13)		2	1 (7)	2 (13)	
3	–	1 (7)		3	–	–	
5	1 (7)	1 (7)		5	–	–	
Apophyseal Injury - N of vertebrae per participant with positive findings							
0	14 (93)	15 (100)	1.00	0	14 (93)	14 (93)	1.00
1	1 (7)	–		1	1 (7)	1 (7)	
Spondylolisthesis - N of vertebrae per participant with positive findings							
0	15 (100)	15 (100)	N/A	0	15 (100)	14 (93)	1.00
1	–	–		1	–	1 (7)	

For categorical variables n (%) is presented

### Reliability measures

Intra- and interobserver reliability measures were high for all spinal parameters. Gwet´s agreement coefficient for interobserver agreement (95% CI) were 0.98 (0.95–1.0) for Modic Type, 0.83 (0.79–0.87) for Endplate defect score, 0.81 (0.75–0.98) for Pfirrmann classification and 0.97 (0.92–1.0) for Schmorl´s nodes. Gwet´s agreement coefficient for intraobserver agreement was 0.98 (0.97–1.0) for Modic Type, 0.79 (0.72–0.85) for Endplate defect score, 0.93 (0.89–0.97) for Pfirrmann classification and 0.97 (0.92–1.0) for Schmorl´s nodules. Analysis of intra- and interobserver reliability for apophyseal injuries and spondylolisthesis was not applicable due to the limited size of the data.

## Discussion

In this MRI-based study there was an overall low occurrence of spinal changes in elite climbers, with non-significant differences compared to healthy controls. In addition, there was a low occurrence of high-grade degenerative changes of the endplates and IVDs.

Considering the lack of previous studies investigating spinal changes among climbers this study offers a first insight into this matter. While degenerative changes of the IVD and endplates were present in the climbing group, the cumulative amount of these changes were not considerable, nor statistically higher than in the control group. Attention has previously been raised with regard to spondylolysis of climbers [28]. While no climbing participant in this study displayed signs of such injury, it should not be concluded that climbing at an elite level carries no risk for sustaining these injuries. Two cases of apophyseal injury were present. Apophyseal injuries, shown to occur among adolescents in sport specific environments [29], seemingly occur at considerably higher rates (up to 16% of all youth injuries) in other sports compared to the present study, indicating that these injuries may not be a typical characteristic of climbing [29].

While climbing, the spine of climbers is under the influence of many different loading patterns. The spinal loads depend on the route climbed, the angle of the climb, and the techniques used. The most obvious situation where axial load is placed on the spine is however when the climber descends from boulders and lands in a squat position to absorb ground reaction forces. This may pose large compressional loads of the spine, commonly combined with flexion. Due to these loading patterns, it was hypothesized that climbers would have a high occurrence of radiographic spinal changes. However, a high occurrence of spinal changes in the present elite climbers could not be verified. These results differ from other studies on physical activities with high spinal loads, such as gymnastics [30], skiing [6], beach volleyball [9] and weightlifting [7], sports in which athletes present with a higher prevalence of spinal changes. A potential difference between climbing and these sports, is the variation in the spinal load in climbing. Whereas aforementioned sports contain repetitive loading patterns, the frequent change in loading patterns during climbing may be a protective factor for adaptations of the spine, even though the loads on the spine are often considerable while climbing.

The low prevalence of radiographic spinal changes could arguably also be attributed to an insufficient accumulation of lifetime load on the participants. The demographics of the climbing group, however, contradicts this. The climbing group had been active climbers for a mean of 12 years, and the yearly training volume was considerable. In addition, 86% of the participants commonly used jumps to descend boulders. Although not examined on an individual level, the mean age, and years training, suggest that participants had been climbing through the growth spurt, a phase in which the athletes are susceptible to spinal injuries [21].

While the association between back pain and changes of the spine, lies outside the scope of this paper, it is worth noting the high one-year prevalence of lumbar back pain in the climbing group (60%). However, minimal disability was associated to the back pain, as measured by the Oswestry disability index. While back pain is common in sport specific environments, the prevalence of back pain varies depending on sport (one-year prevalence of lumbar pain 17–94%) [31] and also on which patient reported outcome measure is used [32]. A systematic review by Farahbakhsh et al. [31], evaluated the one-year prevalence of lumbar back pain in a variety of sports including rowing (33–63%), gymnastics (39%), weightlifting (59%) [31]. The present study had a higher or similar one-year prevalence of lumbar back pain compared to these high spinal load sports. While some studies have addressed acute onset back pain related to specific injury events in climbers [33, 34], no studies have specifically examined the overall prevalence of thoracolumbar back pain or spinal changes on MRI among climbers. The one-year prevalence of thoracic back pain among climbers in this study can also be considered high. In the climbing group, 33% of the participants (13% of controls) reported thoracic back pain which is higher as compared to a 15–28% yearly prevalence among the general population [35].

### Limitations

The small differences in findings between groups of climbers and controls could not be statistically verified due to the limited sample size. Nonetheless, the sample of participants constitute the majority of elite Swedish climbers and as such offers a valuable description of radiographic spinal changes in this specific group of athletes. Similarly, a risk that the study did not include climbers possibly already retired from climbing due to dysfunction at a young age cannot be completely discarded, with potential sampling bias as a result. Potential sampling bias may also be present in the control group, as indicated by the high training loads of some of the control participants.

Furthermore, conventional MRI techniques carry inherent methodological limitations, and are unable to detect subtle spinal changes. Further studies with increased sample sizes and participants of younger and older age are needed to avoid sampling bias and to confirm the results of this study. Despite these limitations, the structured evaluation of both spinal changes and thoracolumbar back pain remains a strength, and the novelty of the study offers new insights.

## Conclusion

In this small cross-sectional study, only a low proportion of elite climbers displayed changes of the spinal endplates or intervertebral discs, as opposed to other sports with high spinal loads. Most observed abnormalities were low grade degenerative changes and did not differ statistically compared to controls.

## Data Availability

The data that support the findings of this study are available from the corresponding author upon reasonable request.

## Abbreviations

IVD: Intervertebral discs
MRI: Magnetic resonance imaging
IRCRA: International rock climbing research association
BMI: Body mass index
